# ALPK1‐Dependent cIAP1 Degradation Regulates *Helicobacter pylori* ‐Induced Apoptosis

**DOI:** 10.1096/fj.202500764R

**Published:** 2025-05-13

**Authors:** Gunter Maubach, Michelle C. C. Lim, Michael Naumann

**Affiliations:** ^1^ Medical Faculty Otto von Guericke University, Institute of Experimental Internal Medicine Magdeburg Germany

## Abstract

*Helicobacter pylori* infection poses a significant risk for disrupting the gastric epithelium by inducing inflammation and apoptosis. Here, we identify alpha kinase 1 (ALPK1) as essential in *H. pylori* ‐induced apoptosis. The absence of ALPK1 leads to the accumulation of cellular inhibitor of apoptosis 1 (cIAP1) upon *H. pylori* infection, which raises the threshold for the induction of apoptosis. Ablation of cIAP1 with a SMAC‐mimetic restores the level of apoptosis induced by *H. pylori* in these cells. Our findings highlight the impact of ALPK1 on the stability of cIAP1, influencing *H. pylori* ‐induced apoptosis.

## Graphical abstract

We have previously shown that ALPK1 is essential for the activation of NF‐κB by *H. pylori* . In ALPK1‐dependent cIAP1 degradation regulates *Helicobacter pylori* ‐induced apoptosis by Gunter Maubach*, Michelle C.C. Lim, Michael Naumann, we demonstrate that in wild‐type epithelial cells, *H. pylori* infection directs cIAP1 degradation. In ALPK1 knockout cells, stabilization of cIAP1 raises the threshold for apoptosis induction. The ablation of cIAP1 using a SMAC‐mimetic increases apoptosis in ALPK1‐deficient cells. Created in BioRender.

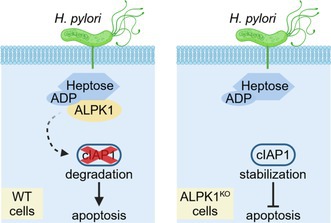

## 1 Introduction

The Gram‐negative bacteria *H. pylori* colonizes the surface of the human gastric epithelium mucosa. Persistent infection with *H. pylori* causes chronic gastritis and can lead to a plethora of gastrointestinal disorders including malignant gastric diseases in a subgroup of individuals [1]. *H. pylori* induces apoptosis in gastric epithelial cells, a distinct form of regulated cell death that is characterized by receptor‐ or cytochrome c release‐mediated activation of caspase‐8/10 or caspase–9, respectively. Both pathways converge on executioner caspases‐3/7, which cleave key substrates, leading to apoptosis [2]. The IAP family of proteins consists of eight members, characterized by the presence of the baculovirus IAP repeat (BIR) domain, which are overexpressed in many cancers [3]. Functionally distinct, they prevent apoptosis through various mechanisms that target caspases‐3/7 and ‐9. Amongst them, only XIAP has been reported to directly inhibit caspases, while cIAPs may partially suppress apoptosis by ubiquitinylating caspases [3].

Intriguingly, when investigating the dual function of ALPK1 and tumor necrosis factor receptor‐associated factor (TRAF)‐interacting protein with forkhead‐associated domain (TIFA) in *H. pylori* ‐induced activation of nuclear factor kappa‐light‐chain‐enhancer of activated B cells (NF‐κB) signaling previously, we observed the rapid turnover of cIAP1 [4]. We did not notice this phenomenon in cells that were deficient in either TIFA or ALPK1. Given that IAPs are established inhibitors of apoptosis, we investigated the role of ALPK1 and cIAP1 in *H. pylori* ‐induced apoptotic signaling. We discovered that the stabilization of cIAP1 in ALPK1‐knockout (ALPK1^KO^) cells elicits less apoptosis upon *H. pylori* infection.

## 2 Materials and Methods

### 2.1 Cell Culture and Bacterial Culture Conditions

Wild‐type (WT) AGS (ATCC CRL‐1739) and ALPK1^KO^ AGS cells were routinely cultivated as described elsewhere [4]. *H. pylori* WT strains P1 and P12 were grown and used for infection as previously described [4]. For cIAP1 stability experiments, cells were treated with 50 μg/mL cycloheximide for 15 min prior to infection. Birinapant (final concentration 100 nM) was added to cells 15 min before infection with *H. pylori* .

### 2.2 Transfection of siRNA

Cells were seeded at a density of 6 × 10^5^ per 60 mm culture dish. Transfection of siRNA was performed as described elsewhere [4]. Cells were infected with *H. pylori* or treated with 10 ng/mL TNF (Peprotech, 300‐01A) after 48 h of siRNA transfection.

### 2.3 Flow Cytometry

Analysis of annexin V/propidium iodide (PI) stained cells for apoptosis was performed as described elsewhere [5]. All experiments were statistically analyzed using One‐Way ANOVA with Bonferroni's post hoc test, and significance levels are: **p ≤ 0.001; ***p ≤ 0.0001; ****p ≤ 0.00001; and n.s. (not significant).

### 2.4 Preparation of Whole Cell Lysates and Immunoblotting

Cells were lysed in cell lysis buffer (50 mM Tris/HCl pH 7.5, 150 mM NaCl, 5 mM EDTA, 10 mM K_2_HPO_4_, 10% glycerol, 1% Triton X‐100, 0.5% NP‐40) supplemented with phosphatase inhibitors (1 mM sodium vanadate, 1 mM sodium molybdate, 20 mM sodium fluoride, 10 mM sodium pyrophosphate and 20 mM 2‐phosphoglycerate), 1 mM AEBSF, and protease inhibitor mix (cOmplete, Mini, EDTA‐free, Roche). Samples were separated by SDS‐PAGE and analyzed by immunodetection as described elsewhere [4].

### 2.5 Densitometric Analysis

The densitometric quantification of band intensities was performed using ImageJ Schneider, C. A., W. S. Rasband, et al. (2012). “NIH Image to ImageJ: 25 years of image analysis.” Nature Methods 9(7): 671–675. The combined data of two experiments were statistically analyzed using One‐Way ANOVA with Bonferroni's post hoc test, and significance levels are: *p ≤ 0.05; ***p ≤ 0.0001 and n.s. (not significant).

## 3 Results

**FIGURE 1:**
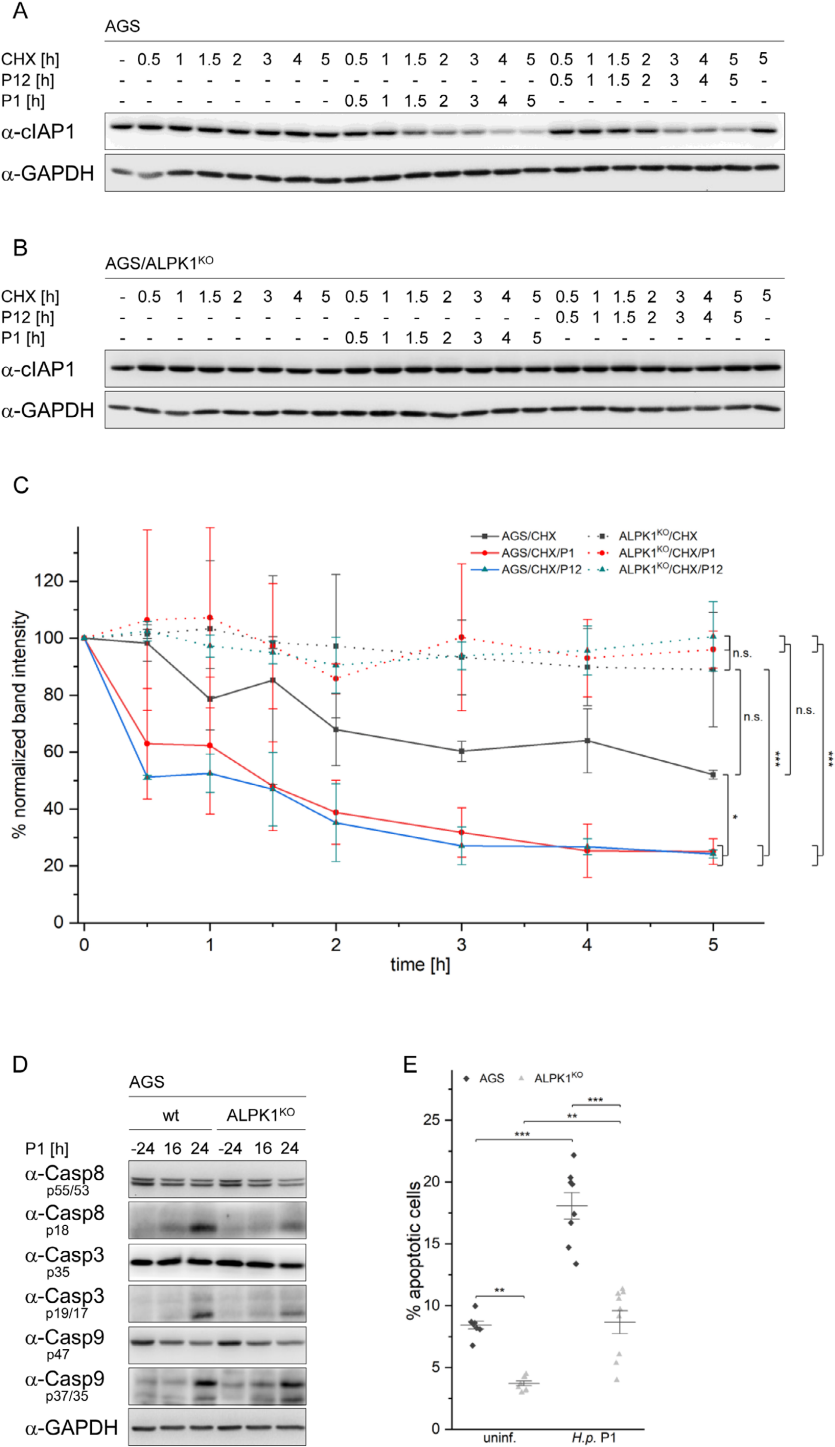
Degradation of cIAP1 by *H. pylori* infection is affected by the absence of ALPK1. (A, B) WT and ALPK1^KO^ AGS cells were pretreated with cycloheximide and left uninfected or infected with *H. pylori* strains P1 and P12. Cell lysates were analyzed by immunoblotting using the indicated antibodies. (C) Intensities of bands in immunoblots were normalized against GAPDH, quantified and plotted against time. (D) WT and ALPK1^KO^ AGS cells were left uninfected or infected with *H. pylori* strain P1. The processing of caspases in cell lysates was analyzed by immunoblotting using the indicated antibodies. (E) WT and ALPK1^KO^ AGS cells were left uninfected or infected with *H. pylori* strain P1 and apoptosis was analyzed using an annexin V/PI assay. *p ≤ 0.05; **p ≤ 0.001; ***p ≤ 0.0001 and n.s. (not significant)

To assess the stability of cIAP1 in uninfected and *H. pylori* ‐infected WT as well as ALPK1^KO^ AGS cells, we treated the cells with cycloheximide to inhibit protein synthesis. We noted an increased turnover of cIAP1 in WT cells upon infection with *H. pylori* strains P1 and P12 (Figure 1A). In ALPK1^KO^ cells, the abundance of cIAP1 remained stable with or without infection (Figure 1B). The statistical analysis of two independent experiments (Figure 1C) showed a significant difference in the turnover of cIAP1 between uninfected and infected WT cells, as well as between infected WT and infected ALPK1^KO^ cells. We observed no significant differences between uninfected and infected ALPK1^KO^ cells, uninfected WT and ALPK1^KO^ cells, as well as uninfected WT and infected ALPK1^KO^ cells (Figure 1C). We corroborated this observation by fitting our data to a first‐order exponential decay model using non‐linear regression to estimate the half‐life of cIAP1 (data available from the corresponding author at request). Next, we examined the processing of caspases and induction of apoptosis in response to *H. pylori* infection in WT and ALPK1^KO^ cells. *H. pylori* infection induced processing of caspases‐8 and ‐3 to different extents in WT and ALPK1^KO^ cells. In contrast, no distinct difference in the processing of caspase‐9 was observed in WT and ALPK1^KO^ cells (Figure 1D). Analysis of apoptosis using an annexin V/PI staining assay and flow cytometry showed a strong reduction in the induction of apoptosis in ALPK1^KO^ cells upon *H. pylori* infection (Figure 1E).

**FIGURE 2:**
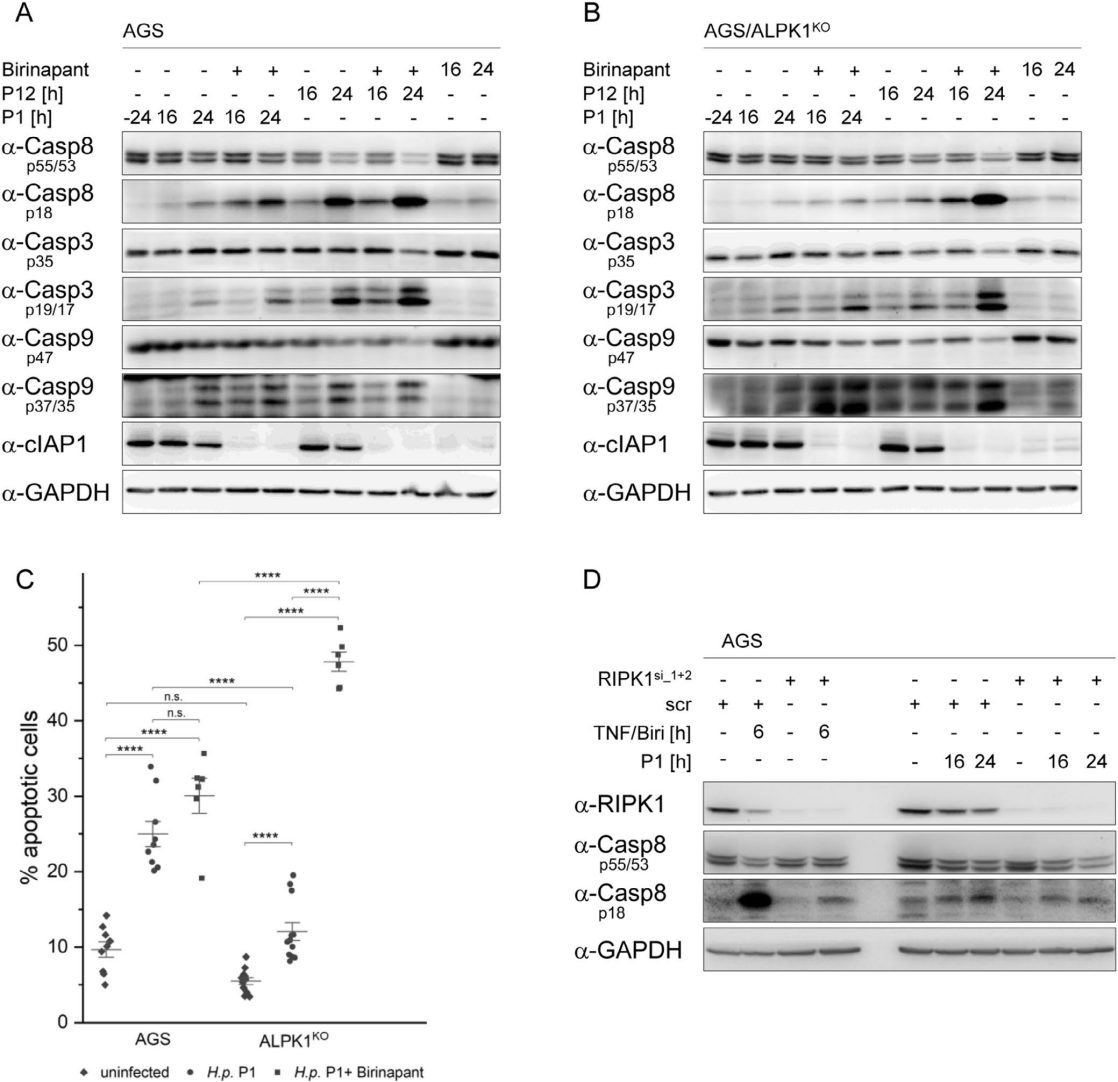
SMAC mimetic‐induced cIAP1 degradation influences the processing of caspases and apoptosis. (A, B) WT and ALPK1^KO^ AGS cells were pretreated with Birinapant and left uninfected or infected with *H. pylori* strains P1 and P12. The processing of caspases in cell lysates was analyzed by immunoblotting using the indicated antibodies. (C) WT and ALPK1^KO^ AGS cells were pretreated with Birinapant and left uninfected or infected with *H. pylori* strain P1 and apoptosis was analyzed using an annexin V/PI assay. (D) WT AGS cells were depleted of RIPK1 using siRNA transfection. Cell lysates were analyzed by immunoblotting using the indicated antibodies. ****p ≤ 0.00001 and n.s. (not significant).

To determine whether the cIAP1 stabilization in ALPK1^KO^ cells contributes to the reduced capacity of *H. pylori* in the processing of caspases and apoptosis, we stimulated the autocatalytic degradation of cIAP1 using the specific SMAC‐mimetic Birinapant. No processing of caspases was observed in cells treated with Birinapant alone, contrary to the anticipated increase in the processing of caspases‐3, ‐8, and ‐9 in both WT and ALPK1^KO^ cells upon *H. pylori* infection (Figure 2A,B). Additionally, apoptosis induction was also affected in both groups of cells, although the difference between Birinapant/ *H. pylori* ‐treated and untreated WT cells was not as striking as in ALPK1^KO^ cells (Figure 2C).

Finally, we investigated whether the loss of receptor‐interacting serine/threonine‐protein kinase 1 (RIPK1), a cIAP1 target reported to be involved in apoptosis [6], affects the processing of caspases. We performed a knockdown of RIPK1 and analyzed caspase‐8 processing after TNF/Birinapant treatment or *H. pylori* infection. In both TNF/Birinapant treatment and *H. pylori* infection groups, we observed a decrease in the processing of caspase‐8, though there was only a slight decrease in *H. pylori‐infected* cells (Figure 2D).

## 4 Discussion

*H. pylori* infection represents a major risk factor for the development of gastric diseases, including gastric cancer. Infection of gastric epithelial cells with *H. pylori* induces a series of cellular processes, one of which is activation of the classical as well as the alternative NF‐κB signaling pathways. Interestingly, these complex processes have an impact on either the induction or suppression of apoptosis [7]. In response to *H. pylori* infection, TIFA becomes phosphorylated by ALPK1 and interacts with TRAF2 in the NIK regulatory complex, leading to the proteasomal degradation of cIAP1 [4]. Here, we observed the stabilization of cIAP1 in cells deficient in the adenosine diphosphate (ADP)‐glycero‐β‐D‐manno‐heptose receptor ALPK1. This is attributed to the missing interaction of TIFA and TRAF2, thereby maintaining a stable association of cIAP1 and TRAF2 in these cells [4]. In ALPK1^KO^ cells, the diminished processing of caspases and apoptosis induction upon *H. pylori* infection can be ascribed to the anti‐apoptotic function of the high levels of cIAP1, which is well documented in the literature [6]. To validate this assumption, we utilized Birinapant to deplete cIAP1 in cells. Herein, we observed the degradation of cIAP1 and the concomitant increase in the processing of caspases and apoptosis.

Due to the complexity of events triggered upon *H. pylori* infection, the induction of caspase processing and apoptosis in AGS cells is detected much later compared to other triggers like FasL, TRAIL, or TNF/Birinapant. The interplay between cIAP1 degradation and the synthesis of anti‐apoptotic gene products upon NF‐κB activation by *H. pylori* infection, both events being absent in ALPK1^KO^ cells, partially accounts for this different threshold in the induction of apoptosis.

In TNF/Birinapant‐treated cells, cIAP1 degradation led to the recruitment of RIPK1 into a complex containing caspase‐8, triggering its processing. This was abrogated in RIPK1‐depleted cells. Upon *H. pylori* infection, the RIPK1‐dependent induction of caspase‐8 processing seems to play a minor role as we observed only a slight reduction of this processing by RIPK1 siRNA treatment. This small change in processing could be explained by ER stress‐mediated cell death in WT cells [8]. In contrast, due to the absence of NF‐κB activation in ALPK1^KO^ cells, the ER stress is diminished, implying that other apoptosis‐inducing mechanisms, influenced by cIAP1, are at play in these cells [9, 10]. All in all, that ALPK1 seems to play a role in the regulation of apoptosis upon *H. pylori* infection is intriguing and warrants further investigation to determine if ALPK1 could be a valid target for preventing gastric mucosal damage.

## Author Contributions

G.M. and M.N. conceived the study. G.M. designed the experiments. G.M. and M.C.C.L. performed the experiments. G.M. analyzed and interpreted the results of the experiments. G.M., M.C.C.L., and M.N. wrote the manuscript.

## Conflicts of Interest

The authors declare no conflicts of interest.

## Supporting information


Figure S1.

## Data Availability

Data will be shared upon request by contacting the corresponding author Gunter Maubach at: gunter.maubach@med.ovgu.de.
