# Supplementary material for: ALPK1‐Dependent cIAP1 Degradation Regulates Helicobacter pylori ‐Induced Apoptosis
Source: FASEB J. 2025 May 13;39(10):e70593. doi: 10.1096/fj.202500764R (PMC12070357; doi:10.1096/fj.202500764R)
Supplement: Supplementary file 1 — Figure S1. [file FSB2-39-e70593-s001.docx]

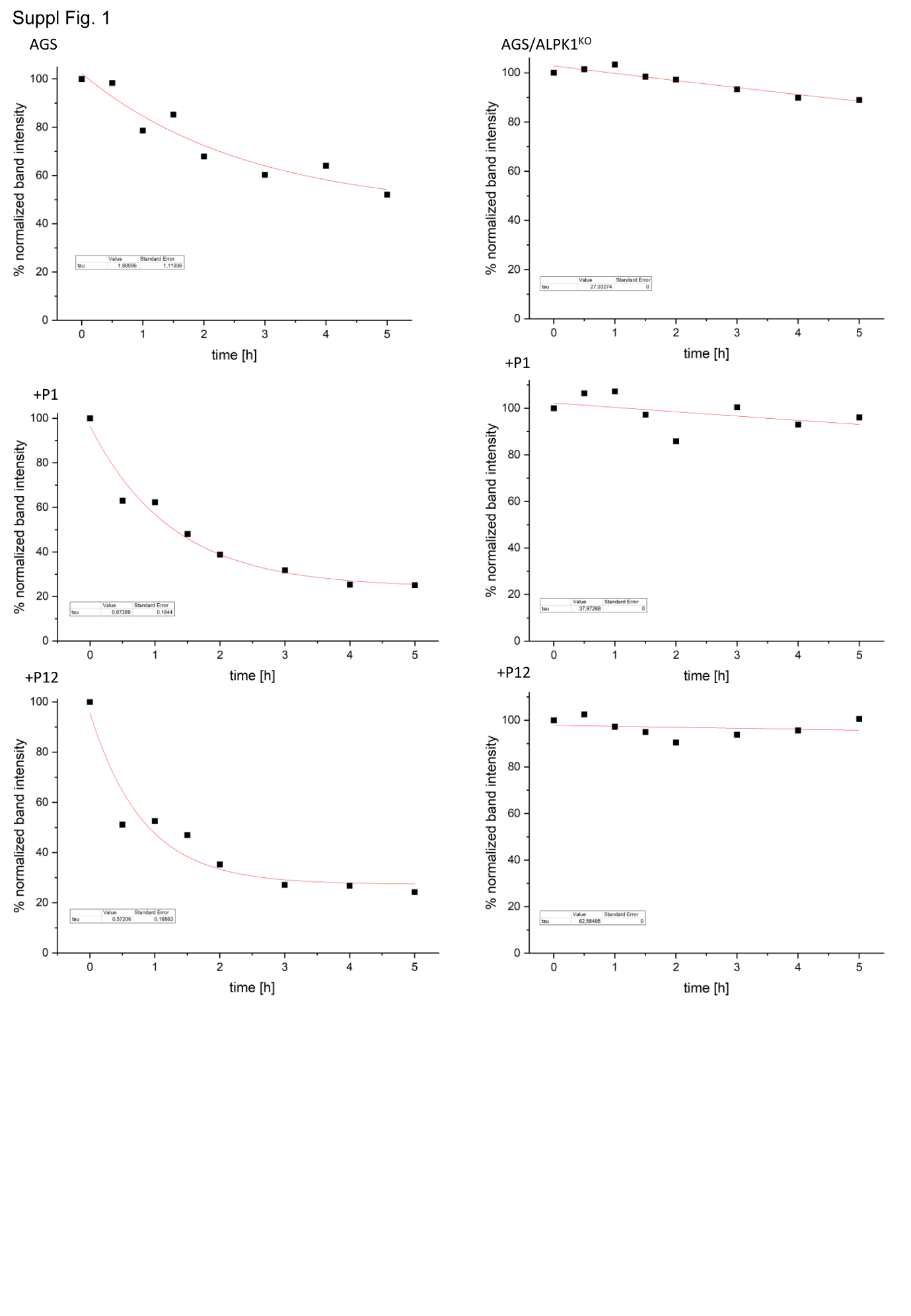


Figure S1 Estimation of the half-life (*τ*) using combined data from the densitometry analysis of two experiments shown in Figure 1. Due to the slow degradation of cIAP1 in ALPK1^KO^ cells, the time constant (*t*1) was estimated ($k=\frac{1}{t1}$) from the degradation rate (*k*), which was calculated from the intensity of the bands at 4 hours in percent using the formula $k=\frac{\ln({I_{0}}/{I_{4h})}}{4h}$. The estimated *t*1 was kept constant during the nonlinear curve fit. The software used was OriginPro 2020b (9.7.5.184).
